# Rabbit ATG/ATLG in preventing graft-versus-host disease after allogeneic stem cell transplantation: consensus-based recommendations by an international expert panel

**DOI:** 10.1038/s41409-020-0792-x

**Published:** 2020-01-22

**Authors:** Francesca Bonifazi, Marie-Thérèse Rubio, Andrea Bacigalupo, Jaap Jan Boelens, Jürgen Finke, Hildegard Greinix, Mohamad Mohty, Arnon Nagler, Jakob Passweg, Alessandro Rambaldi, Gérard Socie, Carlos Solano, Irwin Walker, Giovanni Barosi, Nicolaus Kröger

**Affiliations:** 1 Institute of Hematology “Seragnoli”, University Hospital “S. Orsola Malpighi”, Bologna, Italy; 2 Department of Hematology, Hôpital Brabois, CHRU Nancy and CNRS UMR 7365, Biopole del’Université del Lorraine, Vendoeuvre les Nancy, France; 3 “Fondazione Policlinico Universitario A. Gemelli IRCCS”, Rome, Italy; 4 Institute of Hematology, Università Cattolica del Sacro Cuore, Rome, Italy; 5 Stem Cell Transplantation and Cellular Therapies, MSK Kids, Memorial Sloan Kettering Cancer Center, New York, NY USA; 6 Freiburg University Medical Faculty and Medical Center, Department for Medicine, Hematology, Oncology and Stem Cell Transplantation, Freiburg, Germany; 7 Division of Hematology, Medical University of Graz, Auenbruggerplatz 38, 8036 Graz, Austria; 8 Service d’Hématologie clinique et Thérapie cellulaire, Hôpital Saint-Antoine, Assistance Publique-Hôpitaux de Paris (AP-HP), Sorbonne University, INSERM UMRs 938, Paris, France; 9 Hematology Division, Chaim Sheba Medical Center, Tel Hashomer, Israel; 10 Division of Hematology, Department of Medicine, University Hospital Basel, Basel, Switzerland; 11 Department of Oncology, University of Milano and Azienda Socio Sanitaria Territoriale Papa Giovanni XXIII, Bergamo, Italy; 12 Service d’ Hématologie-greffe, Hôpital Saint-Louis, Assistance Publique-Hôpitaux de Paris, Université Paris Diderot, Inserm UMR 976, Paris, France; 13 Hematology Service, Hospital Clínico Universitario-INCLIVA; Department of Medicine, School of Medicine, University of Valencia, Valencia, Spain; 14 Department of Medicine, McMaster University, Hamilton, ON Canada; 15 IRCCS Policlinico S. Matteo Foundation, Pavia, Italy; 16 University Hospital Eppendorf, Hamburg, Germany

**Keywords:** Immunological disorders, Diseases

## Abstract

This collaborative initiative aimed to provide recommendations on the use of polyclonal antithymocyte globulin (ATG) or anti-T lymphocyte globulin (ATLG) for the prevention of graft-versus-host disease (GvHD) after allogeneic hematopoietic stem cell transplantation (HSCT). A comprehensive review of articles released up to October, 2018 was performed as a source of scientific evidence. Fourteen clinically relevant key questions to the domains indication, administration, and post-transplant management were developed and recommendations were produced using the Delphi technique involving a Panel of 14 experts. ATG/ATLG was strongly recommended as part of myeloablative conditioning regimen prior to matched or mismatched unrelated bone marrow or peripheral blood allogeneic HSCT in malignant diseases to prevent severe acute and chronic GvHD. ATG/ATLG was also recommended prior to HLA-identical sibling peripheral HSCT with good but lesser bulk of evidence. In reduced intensity or nonmyeloablative conditioning regimens, ATG/ATLG was deemed appropriate to reduce the incidence of acute and chronic GvHD, but a higher risk of relapse should be taken into account. Recommendations regarding dose, application, and premedication were also provided as well as post-transplant infectious prophylaxis and vaccination. Overall, these recommendations can be used for a proper and safe application of polyclonal ATG/ATLG to prevent GvHD after allogeneic HSCT.

## Introduction

Graft-versus-host disease (GvHD) is one of the most important factor limiting the success of allogeneic hematopoietic stem cell transplantation (HSCT) [1, 2] because it negatively affects both the duration and quality of life of patients after transplant [3].

The most widely used strategy for GVHD prevention, at least in Europe [4], is the addition of rabbit antilymphocyte serum to the standard prophylaxis with a calcineurin inhibitor and either methotrexate or mycophenolate mofetil. There are several formulations of polyclonal antilymphocyte sera available in different countries, generated in animals (rabbits, horses, and pigs) by inoculation of human thymocytes or human cell line. Available rabbit polyclonal antilymphocyte sera are the antithymocyte globulin (ATG) (Sanofi Genzyme, Cambridge MA), derived from rabbit vaccination with human thymocytes, and the anti-T-lymphocyte globulin (ATLG) (Neovii, Rapperswil, Switzerland, formerly ATG Fresenius®), derived from the human Jurkat T-cell line. Horse ATG (hATG, ATGAM®, Pfizer Inc NY, US) is used as first-line therapy of moderate–severe aplastic anemia [5] as well as GvHD prophylaxis [6]. Finally, porcine ATG is also used for the treatment of severe aplastic anemia especially in China and India [7] and, less frequently, in the setting of HSCT [8]. Here we restrict the consensus to rabbit sera because of their prevalent use as drugs for GvHD prevention.

Since rabbit polyclonal serum reacts against host and donor lymphocytes, the expected effects are both the reduction of the risk of GvHD and the prevention of graft failure.

Data regarding the use of rabbit polyclonal serum in patients undergoing allogeneic HSCT indicate that both ATG and ATLG significantly reduce the incidence of GvHD but their administration is associated with delayed immune reconstitution [9, 10]. As a consequence, they lead potentially to an increased risk of infections and of leukemia relapse. Despite the most recent meta-analysis did not show any significant benefit of ATG/ATLG on post-transplant survival [11], data from different studies are still discordant because of different formulations, doses, and timing used as well as the heterogeneity of the studied populations, the hematopoietic stem cell sources, and the intensity of the conditioning regimens.

The aim of this collaborative initiative is to provide consensus-based recommendations for indication and administration of ATG/ATLG as part of the conditioning regimen prior to allogeneic HSCT, as well as for posttransplant management. These recommendations should be used as practical guidelines for ATG/ATLG therapy and in helping to address the scientific questions for clinical trials in the domain of indications and administration in allogeneic HSCT.

## The consensus process

Two chairpersons (NK and FB) appointed a panel of 14 physicians (hereafter referred to as the Panel), selected for their expertize in research and clinical practice of allogeneic HSCT. A clinician with expertize in clinical epidemiology (GB) assured the methodological correctness of the process (Delphi technique [12]). The key questions were ranked according to their priority votes, with the 14 that ranked highest forming the set of questions in the present recommendations (Table 1). Searches of the literature in English were performed using MEDLINE, EMBASE, and PubMed database (up to October, 2018). Three panelists drafted statements that addressed the identified key questions, and remaining panelists scored their agreement with those statements and provided suggestions for rephrasing.

**Table 1:** List of key questions.

Domain 1—Indications on use of ATG/ATLG	
1	For which donor type, stem cell source, and type of conditioning of allogeneic HSCT is ATG/ATLG administration indicated, contra indicated, or uncertain?
2	For which type of disease and phase of disease is ATG/ATLG administration during allogeneic HSCT conditioning indicated, contra indicated or uncertain?
3	Are the indications of ATG/ATLG administration during allogeneic HSCT conditioning different according to patient’s age and for pediatric and adult patients?
Domain 2—ATG/ATLG administration protocol	
1	How should side effects of ATG/ATLG administration (such as systemic inflammation response syndrome—SIRS) be prevented?
2	Which is the recommended schedule (timing and doses) of ATG/ATLG administration during myeloablative, reduced intensity, and nonmyeloablative conditioning regimens?
3	What are the factors affecting the choice of the type and the brand of ATG/ATLG?
4	Should doses, schedule, and brand of ATG/ATLG administration differ for second transplants?
5	What is the recommended action in case of severe allergic reaction after first dose of ATG/ATLG?
6	Should the administration of ATG/ATLG be modified in case of suspected infection?
Domain 3—Post-transplant management in patients who received ATG/ATLG administration	
1	What post-transplant infection prophylaxis is recommended after ATG/ATLG administration?
2	What post-transplant vaccination is recommended after ATG/ATLG administration?
3	Should DLI indication and dose be modified for patients who received ATG/ATLG?
4	Is universal prophylaxis or monitoring/preemptive treatment of post-transplant lymphoproliferative disease appropriate in patients who received ATG/ATLG?

*HSCT* hematopoietic stem cell transplantation, *ATG* antithymocyte globulin, *ATLG* anti-T-lymphocyte globulin.

## Results

### Domain 1: indications for ATG/ATLG therapy

#### Recommendations

*ATG/ATLG is recommended as part of chemotherapy- or total body irradiation (TBI)-based myeloablative conditioning (MAC) regimen prior to matched or mismatched unrelated bone marrow (BM) or peripheral blood (PB) allogeneic HSCT in malignant diseases to prevent severe acute and chronic GvHD.*

*The Panel also agreed that ATG/ATLG should be recommended prior to HLA-identical sibling PB allogeneic HSCT, even though the evidence of efficacy is based only on one randomized trial, compared with four trials in unrelated allogeneic HSCT.*

*In reduced intensity conditioning (RIC) or nonmyeloablative (NMA) conditioning regimens, the use of ATG/ATLG is appropriate to reduce the incidence of acute and chronic GvHD, but a higher risk of relapse should be taken into account. The Panel agreed that the use of ATG/ATLG in this setting should be considered in relation to the intensity of conditioning and based on risk of disease relapse.*

*The Panel did not reach a consensus on the use of ATG/ATLG as GvHD or graft failure prophylaxis in T-cell replete haploidentical allogeneic HSCT when post-transplant cyclophosphamide (PTCy) is used. Almost all non-PTCy regimens include ATG/ATLG therapy, so no comparative evidence can be derived and these regimens should be followed as originally reported.*

*The Panel did not reach a consensus about the appropriateness of use of ATG/ATLG in cord blood HSCT.*

*ATG/ATLG is recommended as GvHD and graft failure prophylaxis in nonmalignant diseases such as aplastic anemia where no graft-versus-leukemia (GVL) effect is required.*

*The Panel agreed that the indication for ATG/ATLG should not depend on patient age, although the tolerability of ATG/ATLG in older patients is lower.*

#### Summary of evidence

We analyzed five randomized clinical trials (RCTs) evaluating the use of ATG/ATLG in allogeneic HSCT from unrelated (four trials) and related donors (one trial) [13–17]. In the trials with unrelated donors [13–16] the difference in incidence of cGvHD between ATG/ATLG and no ATG/ATLG arms ranged from 11 to 28%, while for acute GvHD (aGVHD), considering the appropriate doses of the drug, it ranged from 15 to 42%. In the study [17] focused on matched related donors, both the incidence and severity of cGVHD decreased with the addition of ATLG.

All the four recent meta-analyses on ATG/ATLG [11, 18–20] found a significant reduction of both cGvHD and aGvHD, without any differences in OS and non-relapse mortality (NRM), but one reported a higher risk of relapse [11]. There were discrepancies among meta-analyses in results on infections and relapse. In one [16] of the RCTs an increase in relapse after ATLG was reported, particularly in patients receiving a TBI-based conditioning regimen. On the contrary, in a previous trial using the same doses of ATLG (60 mg/kg), no significant increase in relapse was seen [14].

Data from the meta-analyses did not allow the assessment of the impact of RIC on the ATG/ATLG effect on GvHD and risk of relapse. In fact, of the five [13–17] only one [17] included also RIC transplants. Retrospective or nonrandomized studies have reported conflicting results on the impact of ATG/ATLG in the setting of RIC transplants. In particular, a higher dose of ATG/ATLG have been associated with a higher risk of relapse thus leading to a decreased disease-free survival (DFS) [21–24], especially for advanced diseases. On the contrary, in a study of the European Society for Blood and Marrow Transplantation (EBMT) [25] assessing the outcome of patients with acute myeloid leukemia (AML) and undergoing PB transplantation from HLA-identical siblings after a RIC in first complete remission, the use of ATG was not significantly associated with a higher risk of relapse; the outcomes of NRM, leukemia-free survival, and OS were not significantly associated with the use of ATG. Patients with advanced or active disease were included only in some of the RCTs [13–15]: this condition was generally found to be a significant prognostic factor for relapse and for survival, but not related to ATG/ATLG administration. The only available subgroup analysis [26], focused on relapse risk and the use of ATLG in patients transplanted in the advanced phase, showed a non-significant interaction between disease phase and ATLG treatment in the setting of unrelated transplants.

In T-cell replete haploidentical HSCTs, ATG at higher doses can be used as GvHD prophylaxis [27–29], but no comparisons with different GvHD prophylaxis regimens are available. In T-cell deplete haploidentical HSCT, ATG was combined with TCR-αβ T-cell depletion to reduce both the risk of graft failure and GVHD [30, 31]; with a TCR-α/β depleted graft, a negative effect of ATG given close to transplantation is expected since it may reduce the GvL effect played by NK cells and gamma/delta T cells.

In cord blood HSCT, the addition of ATG/ATLG resulted in a lower survival rate [32–35], although there is a suggestion that results could be improved with earlier timing and/or lower doses of ATG [36, 37].

Specifically, in the pediatric setting, lower doses of ATLG resulted in comparable prevention of GvHD as higher ATLG doses but with a significant survival benefit because of lower viral reactivation rate [38].

Finally, in non-malignant diseases, the role of ATG/ATLG is easier to promote because GvHD should be completely abrogated and an effective prevention of graft failure is also required. Although not yet confirmed in the context of RCTs, which failed in the past to demonstrate an advantage using ATG [39], a recent report from the EBMT Severe Aplastic Anemia Working Party, with large numbers of patients, confirms the beneficial role of in vivo T-cell depletion with ATG or Alemtuzumab, which was associated with a significant survival benefit [40, 41].

### Domain 2—ATG/ATLG administration protocol

#### Recommendations

*In order to mitigate infusion reactions of ATG/ATLG, premedication with steroids and antihistamine, or acetaminophen and a long-lasting infusion (≥12 h) in a central high flow catheter are highly recommended.*

*The performance of skin tests before infusion to anticipate severe allergic reactions is not recommended.*

*In the event of reactions, it is suggested to temporarily discontinue ATG/ATLG infusion, to strengthen the premedication (higher doses of steroids, antihistamine, or acetaminophen) and to increase the time of infusion, given that patients should be carefully (or intensively) monitored. In the very rare event of severe reactions (e.g., cytokine release syndrome grade 4) a permanent discontinuation is justified.*

*Reduction of ATG/ATLG dose for a suspected infection occurring during the conditioning time should be based on an individual patients’ case.*

*Recommended doses during MAC are 30 mg/kg and 60 mg/kg of ATLG for sibling and unrelated transplants, respectively, from days −3 to −1 and 4.5–7.5 mg/kg for ATG. For children, ATLG dosing at 15 mg/kg is as effective as 30 mg/kg and has been demonstrated to be associated with better event-free survival.*

*In the absence of prospective controlled studies on ATG/ATLG in the setting of RIC and NMA transplants, no recommendation on doses can be given.*

*No factors affecting the choice of the brand of rabbit polyclonal globulin can be advised. The choice should be preferably based on center experience.*

*The doses of ATG/ATLG should be calculated according to body weight, although a recent study suggests that optimal ATG/ATLG exposure may be improved when based on the absolute number of lymphocytes (ALC).*

*Second transplants should be carried out with similar doses of both ATG/ATLG as first transplants.*

#### Summary of evidence

ATLG/ATG administration can be complicated by several infusion reactions such as fever, chills, erythema, dyspnea, oxygen desaturation, nausea/vomiting, diarrhea, abdominal pain, hyperkalemia, tachycardia hypotension or hypertension, malaise, rash, urticaria, headache, arthralgia, myalgia, hepatic cytolysis, and hypotension (level of up to severe), that can be even unresponsive to conventional vasopressors or, although rarely, systemic anaphylaxis. Most symptoms can be attributed to the cytokine release syndrome (CRS) and are reversible. CRS and other forms of infusion reactions cannot be anticipated (e.g., by epicutaneous or intradermal test). Serum sickness can develop after 5–15 days and it generally responds well to steroid treatment [42–47].

Dose and timing of administration of ATLG and ATG varies substantially among transplant centers. For ATLG the total dose varies from total doses of 15–60 mg/kg; conventional timing is generally from days −3 to −1 but also from days −6 to −2 or −4 to −2 are reported. The ATG dose varies from 4.5 to 7.5 mg/kg, with a schedule of administration from days −3 to −1, or −2 to +1, and −4 to −2. In the real life, doses of ATG/ATLG are different from those used in clinical trials, as demonstrated by single-center experiences and nonrandomized studies [48]. In the randomized setting, three studies compared two doses of ATG/ATLG: Bacigalupo et al. [13] employed two different doses of ATG (15 mg/kg or 7.5 mg/kg) in two separate randomized trials with MAC conditioning in BM transplantation from unrelated donors: rates of aGvHD appeared worse (69%) with the lower dose as compared with the higher (37%). However, the higher dose was associated with more frequent serious infections. This trial was conducted before high resolution HLA typing was available and GvHD may have been more severe. In the pediatric setting [38], a lower dose of 15 mg/kg (experimental arm) of ATLG given to children suffering from high risk acute leukemia and undergoing unrelated transplants appears to be beneficial as compared to 30 mg/kg (control arm) in terms of GVHD prevention, while the event-free survival was superior in the experimental arm because of the lower incidence of viral infections. No significant difference between two different doses of ATG (6 mg/kg vs. 10 mg/kg) in a RCT focused on T-replete haploidentical [27] has been shown, in terms of DFS, but higher doses were associated with an increased incidence of Epstein–Barr virus (EBV) reactivation (25.3% vs. 9.6%). Of note in all the three studies [13, 27, 38] higher doses were always associated with higher infectious complications but not with recurrence of the original disease, with the exception of one study [13], which used very high doses of ATG (15 mg/kg).

A non-randomized comparison between 60 mg/kg vs. 30 mg/kg of ATLG [49], in patients with hematological malignancies receiving a MAC regimen, showed that higher doses were associated with an inferior outcome because of greater fatal infections, while aGvHD, cGvHD, and relapse were similar in the two groups. Binkert et al. [50] reported a significant reduction of viral infections, cGvHD, NRM, and increased OS after 35 mg/kg of ATLG in comparison with a higher standard dose (60 mg/kg) and no ATLG, in the setting of related and unrelated transplants. Retrospective analyses reported a significant decrease of cGvHD and less commonly aGVHD even with very low doses of ATLG (15–30 mg/kg) in several series of patients transplanted after MAC regimen, mainly with PBSC, both from matched related and unrelated donors [51–54].

Regarding ATG, Ravinet et al. [55] reported that the addition of ATG after a MAC regimen in patients with acute leukemia and MDS transplanted from a well-matched unrelated donor (10/10) was found to significantly reduce aGvHD and cGvHD, and improve graft-versus-host disease-free, relapse-free survival only after PBSC but not after BM transplantation. Survival, relapse, or NRM were not affected by ATG administration.

Ratanatharathorn et al. [56] reported significantly lower NRM, cGvHD incidence, and severity in patients receiving 4.5 mg/kg of ATG retrospectively compared with those who did not receive ATG, independently from the intensity of the conditioning regimen. The problem of dosage is particularly unsolved in the context of RIC transplants where the balance between the intensity of conditioning regimen and GVL–GvHD risk is sensitive [57]. For this reason, in the absence of a focused clinical trial, the Panel agreed to take a dose reduction of ATG/ATLG into consideration. Devillier et al. [58] reported favorable outcomes (GvHD prevention, no relapse increase) using ATG at 5 mg/kg in patients with AML and MDS receiving PBSC from both a matched related donor and unrelated after a RIC regimen [59]. The EBMT reported a nonsignificant increase of relapse when ATG dose was <6 mg/kg in patients that had a matched related donor [60]. Currently used doses of ATG/ATLG are calculated according to body weight [13–17]. Calculating the ATG/ATLG dose according to cellular target, such as ALC before infusion of the first dose [61, 62], which has been shown to provide optimal drug exposure and then to maximize the benefit (GvHD decrease) over the risks (increase in relapse and infection). This concept has been further validated by a *post hoc* analysis of a RCT [16], where those patients with a lower ALC (<0.1 × 10^9^/L) at the time of first ATLG infusion, the progression free and OS was inferior in comparison to the placebo arm and that a TBI-based regimen was correlated with a lower ALC, thus increasing the unfavorable effects of ATG.

### Domain 3—posttransplant management in patients who received ATG/ATLG

#### Recommendations

*Post-transplant infection prophylaxis or preemptive therapy for patients receiving ATG/ATLG should be similar to that usually recommended after allogeneic HSCT (against herpes simplex virus (HSV), varicella-zoster virus (VZV), cytomegalovirus, pneumocystis jiroveci, toxoplasma gondii, pneumococcus, and fungi).*

*Post-transplant vaccination schedule for patients receiving ATLG or ATG should be similar to that commonly recommended after allogeneic HSCT.*

*Donor lymphocyte infusion indication and dose should not be modified in patients receiving ATG/ATLG*.

#### Summary of evidence

The spectrum of antigens recognized by ATG/ATLG is wide and includes antibody specificities against T, B lymphocytes, antigen presenting cells, monocytes, and more generally inflammatory cells leading to a variety of mechanisms of action [44, 63–67]. Compared with patients not receiving ATG/ATLG, those treated with ATG/ATLG show slower reconstitution of CD4+ and CD8+ T cells in the PB for at least 1 year after transplantation, in particular in terms of naive and memory CD4+ T cells [8, 9, 50], less importantly of CD8+ cells [14], while the effects on B lympocytes and natural killer cells are still controversial [9, 10, 16, 50].

Impaired T cell reconstitution after allogeneic HSCT is associated with worse survival [68] as well as a higher risk of opportunistic viral reactivations and infections, intracellular bacteria, and fungal infections [69]. In addition to antiinfection prophylaxis [70], patients receiving allogeneic HSCT needed to be vaccinated with attenuated vaccines [71].

The association between in vivo T-cell depletion with ATG/ATLG and increased risks of CMV and EBV reactivation is still controversial mainly because different doses and settings jeopardize the net final effect. From the point of view of mechanism of action, ATG/ATLG administration, causing a delayed immune reconstitution, increases the risk of infections [72, 73]; however not all the studies show an increase in viral infections because of the current dose variation. Both prospective [13–17, 38] and retrospective data [47] confirmed an overall trend toward an increased infection risk only in patients treated with higher doses, while the risk of CMV and EBV reactivation was increased in one meta-analysis [18] but not in another one [19]. It has been suggested that the risk of EBV/PTLD increases in patients with high serum levels of active ATG at the time of transplantation [62], which is consistent with the impact of ATG exposure analyzed by pharmacokinetics on CD4+ immune reconstitution [61, 68]. EBV reactivation can be prevented or preemptively treated with rituximab [74]. Prevention of EBV reactivation my be considered particularly in patients with SAA, because of the combination of risk factors [75].

## Discussion

Clinical trials have documented the efficacy of ATG/ATLG to prevent acute and cGvHD after allogeneic HSCT for malignant and non-malignant diseases. However, a number of practical issues regarding the use of ATG/ATLG are still unsolved, due to the interplay of a large number of confounding variables impacting the outcome after HSCT, making the interpretation of data on the efficacy of ATG/ATLG as GvHD prophylaxis troublesome. In this paper, experts in the field of allogeneic HSCT aimed to use a consensus method, provide recommendations regarding the indication and management of ATG/ATLG before allogeneic HSCT as well as posttransplant monitoring.

ATG/ATLG is now frequently used as GvHD prevention in Europe [4] with the highest evidence derived from RCTs, numerous non-RCTs, and meta-analyses.

ATG/ATLG effectively prevents GvHD, in particular cGvHD, after transplants from almost any donor types; the strongest evidence of efficacy comes from studies on transplants with unrelated and matched related donors (Table 2). Consequently, in these settings the Panel reached a high level of agreement. The increase in infection risk was overall less than expected and if at all, was limited to the use of higher doses of ATG/ATLG. Similarly, a dose-dependent relationship can be found with the speed of immune reconstitution. Relapse is increased only in one [16] of the randomized studies [12–15, 17, 38], in a particular subset of patients receiving a TBI-based conditioning regimen, but a retrospective analysis of the Acute Leukemia Working Party of the EBMT [76] did not confirm this finding. Advanced phase at transplant is a well-recognized risk factor for poorer survival, in particular for relapse, independently of ATLG infusion [26]. ATG/ATLG, in reducing GvHD, could decrease the protective effect of GvHD on relapse, in particular of cGvHD [77]; in this setting, due to the higher risk of relapse a potential further increase in disease recurrence, by means of ATLG infusion, cannot be completely excluded. Accordingly in the real life we observe a clear tendency to use doses lower than those used in the RCTs [78].

**Table 2:** Final recommendations.

Conditioning regimen	Stem cell source	Donor	Recommendation	Agreement among experts
**MALIGNANT DISEASES**				
Myeloablative	Bone marrow/peripheral blood	Unrelated	Recommended	Full
Myeloablative	Peripheral blood	HLA-identical sibling	Recommended	Partial
RIC/NMA	Bone marrow/peripheral blood	HLA-identical sibling/matched or mismatched unrelated	Partially recommended	Partial
Any conditioning plus post-transplant cyclophosphamide	Bone marrow/peripheral blood	Haploidentical	Undecidable	Full
Any conditioning without post-transplant cyclophosphamide)	Bone marrow/peripheral blood	Haploidentical	Advised to follow the conditioning published protocols	Full
Any conditioning	Cord blood transplant	Cord blood	Undecidable	Full
**NON-MALIGNANT DISEASES**				
Any conditioning	Any stem cell source		Recommended	Full

*RIC* reduced intensity conditioning, *NMA* nonmyeloablative conditioning.

Lack of relevant clinical trials specifically addressing critical questions on the indication and use of ATG/ATLG has been highlighted by the experts of this project. A major issue was ATG/ATLG dose optimization. Up to now, no dose finding studies have been performed; moreover, the two formulations (ATLG and ATG) show different pattern of antibody specificity [79], hence results obtained with one globulin cannot be applied to the other one. One possible solution could be to use ATG/ATLG according to pharmacokinetics models, which should be validated in the context of prospective RCTs to properly tailor the doses (and the systemic exposure) to the right intensity of GvHD prophylaxis according to all the factors known to affect prognosis (such as disease, phase, age, HSC sources, and HLA mismatch), in order to counteract the potential negative effects (relapses, infections, and delayed immune reconstitution). The use of pharmacokinetic parameters and the ALC, already performed in retrospective analyses [58, 59] and in a post hoc analysis of a RCT [16], deserve further evidences, possibly in a context of large prospective RCTs, for both ATG and ATLG.

The weaker recommendation issued by the Panel (Table 2) in patients transplanted with an HLA-identical donor mainly derives from a limited evidence available. Only one trial [17] has been carried out and showed the efficacy of ATLG. Even if ATG/ATLG administration was not associated with survival gain, the profound reduction of severe cGvHD significantly enhanced quality of life [80], a fact which cannot be ignored in the patients counseling

High uncertainty resulted in the use of ATG/ATLG in T-cell replete haploidentical transplants when PTCy was used, because of a lack of focused trials (Table 2). It could be one of the most interesting setting for an RCT with the addition or not of ATG/ATLG, in particular when in the context of PB transplantation.

Furthermore, the Panel did not reach consensus on the appropriateness of use of ATG/ATLG in cord blood transplant (Table 2), the use of which has sensibly been decreasing in the last years. The peculiar immunological reconstitution after CB HSCT and the lower number of cellular targets for ATG/ATLG (i.e., lymphocytes of the graft) suggest targeting a lower ATG/ATLG exposure to optimize the negative and positive effects of ATG/ATLG.

Finally, the Panel did not recommend any particular formulation of polyclonal serum, leaving the choice to the investigator’s discretion and personal experience. Head to head comparison between the two brands was claimed as the only possible way to prove overall superiority of one of them.
